# Eps15R and clathrin regulate EphB2‐mediated cell repulsion

**DOI:** 10.1111/tra.12531

**Published:** 2017-11-06

**Authors:** Emma Evergren, Neville Cobbe, Harvey T. McMahon

**Affiliations:** ^1^ Medical Research Council Laboratory of Molecular Biology Cambridge UK; ^2^ Centre for Cancer Research and Cell Biology Queen's University Belfast Belfast UK

**Keywords:** clathrin, EphB2, Eps15, Eps15R, Numb, trans‐endocytosis

## Abstract

Expression of Eph receptors and their ligands, the ephrins, have important functions in boundary formation and morphogenesis in both adult and embryonic tissue. The EphB receptors and ephrinB ligands are transmembrane proteins that are expressed in different cells and their interaction drives cell repulsion. For cell repulsion to occur, trans‐endocytosis of the inter‐cellular receptor‐ligand EphB‐ephrinB complex is required. The molecular mechanism underlying trans‐endocytosis is poorly defined. Here we show that the process is clathrin‐ and Eps15R‐mediated using Co115 colorectal cell lines stably expressing EphB2 and ephrinB1. Cell repulsion in co‐cultures of EphB2‐ and ephrinB1‐expressing cells is significantly reduced by knockdown of Eps15R but not Eps15. A novel interaction motif in Eps15R, DPFxxLDPF, is shown to bind directly to the clathrin terminal domain in vitro. Moreover, the interaction between Eps15R and clathrin is required for EphB2‐mediated cell repulsion as shown in a rescue experiment in the EphB2 co‐culture assay where wild type Eps15R but not the clathrin‐binding mutant rescues cell repulsion. These results provide the first evidence that Eps15R together with clathrin control EphB/ephrinB trans‐endocytosis and thereby cell repulsion.

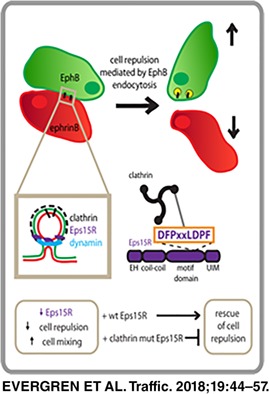

## 1 INTRODUCTION

EphB receptors are a large family of receptor tyrosine kinases that interact with the transmembrane ephrinB ligands. EphB/ephrinB signalling is important for contact‐mediated cell repulsion and tissue patterning during development, but also in adult tissue to establish morphological borders in the intestine or to direct growing axons in the nervous system.^1^, ^2^, ^3^ Furthermore, aberrant expression or mistargeted expression of Eph receptors is associated with cancer cell invasion in prostate, breast and colon cancer.^4^, ^5^, ^6^, ^7^, ^8^, ^9^ The adhesive interaction between EphB receptors and ephrinB ligands activates intracellular signalling pathways that regulate cell‐cell repulsion, migratory behaviour, adhesion and cell polarity. The EphB receptor and the ephrinB ligands are not expressed in the same cell and their interaction can therefore lead to cell‐cell repulsion. However, in order for the cells to repel the EphB/ephrinB complex needs to be physically removed or to dissociate. There are two mechanisms described to date: trans‐endocytosis (trans‐cellular internalisation event) of the receptor‐ligand complex into one of the cells,^10^, ^11^ or cleavage of the extracellular domain by a protease.^12^, ^13^, ^14^, ^15^ The endocytosis of EphB/ephrinB complexes is an unusual type of endocytosis where two trans‐membrane proteins from neighbouring cells are internalised into one cell, thus forming vesicles from two plasma membranes.^11^ The process, called trans‐endocytosis, is dependent on dynamin scission and actin polymerisation but no association with either clathrin or caveolae has been found.^10^, ^11^, ^16^, ^17^ Thus, the molecular mechanism requires further investigation.

The EphB2 receptor interacts with the endocytic adaptor protein Numb.^18^ Numb is a phosphotyrosine binding adaptor protein that regulates receptor trafficking.^19^ Numb interacts directly with the endocytic adaptor protein AP2 and endocytic accessory proteins Eps15/R and intersectin1/2.^20^, ^21^, ^22^ AP2 is the main adaptor protein that directs the clathrin coat formation.^23^ The interaction of Numb with AP2 is mediated by a single DPF (aspartic acid, proline, phenylalanine) motif, an interaction that is too weak to functionally engage the AP2 complex and actively promote clathrin‐mediated endocytosis,^22^, ^23^, ^24^ suggesting that the recruitment of endocytic accessory proteins is important for Numb‐mediated EphB2 receptor endocytosis.

In this study, we identified novel components of the trans‐endocytosis pathway for EphB/ephrinB internalisation. Here we co‐cultured colorectal cancer cell‐lines, Co115, stably expressing the full‐length transmembrane EphB2 receptors or ephrinB1 ligands. Using the co‐culture system, we found that EphB2‐mediated cell‐cell repulsion is a clathrin‐ and dynamin‐dependent mechanism. Moreover we identified a key component in the trans‐endocytosis protein complex, Eps15R, that interacts with EphB2 via the adaptor protein Numb. Using shRNA knockdowns, morphological analysis, and motif mapping, we identified a novel non‐canonical clathrin‐binding motif in Eps15R that was functionally important for EphB2‐mediated cell‐cell repulsion. These results suggest that Eps15R and clathrin‐mediated trans‐endocytosis of the EphB2/ephrinB1 complex is an important mechanism for terminating this adhesive interaction and turning it into cell‐cell repulsion.

## 2 RESULTS

### 2.1 EphB2‐mediated cell repulsion is regulated by clathrin‐mediated endocytosis

**Figure 1:**
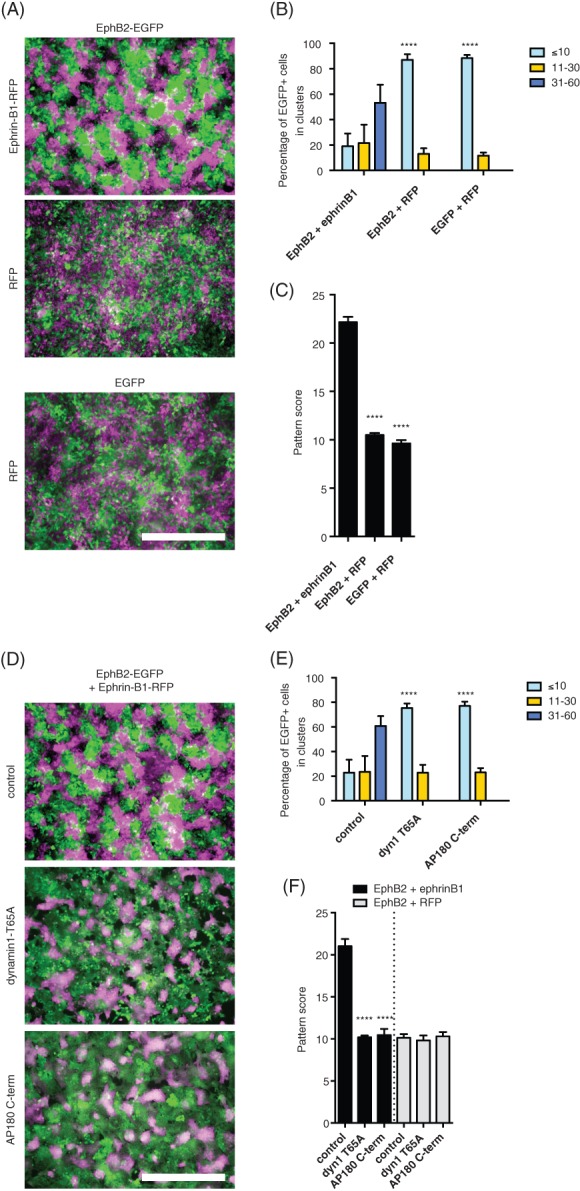
**Trans‐endocytosis of EphB2/ephrinB1 is clathrin‐ and dynamin‐dependent.** *A,* Fluorescent images of co‐cultures of Co115 cells stably expressing EphB2 (EGFP), ephrinB1 (RFP), EGFP or RFP. RFP has been pseudocoloured in magenta for visualisation purposes. Co‐culture of EphB2 (EGFP) and ephrinB1 (RFP) expressing cells resulted in repulsion and clustering of the respective cell lines in a pattern, while the controls showed random cell mixing. Scale bar, 500 μm. *B*, Cell mixing was quantified by counting the percentage of EGFP‐positive cells forming clusters of various sizes. In the absence of ephrinB1‐expressing cells the majority of cells are found in clusters containing <10 cells. Co‐cultures of EphB2 and ephrinB1 Co115 cell lines showed a large proportion of cells in homogenous clusters >30 cells. Error bars represent standard deviation (*n* = 5 random fields per condition). The frequency of cells in small clusters (<10 cells) is a representation of cell mixing and there is a significant difference between EphB2 co‐cultured with ephrinB1 and RFP, and control EGFP plus RFP co‐cultures (****P < 0.0001, Student's two‐tailed unpaired *t* test). *C*, The EphB‐mediated patterning was analysed across random field of co‐cultured EphB2‐ and ephrinB1‐expressing cells. The pattern score takes into account random variation of the proportion of the EGFP‐ and RFP‐expressing cells. Data represent mean ± standard error of the mean. ****P < 0.0001, Student's two‐tailed unpaired *t* test, *n* = 20 per condition in four independent experiments. *D,* Endocytosis was inhibited by expression of two reagents that have established dominant negative impact on endocytosis, dynamin1‐T65A and AP180 C‐terminus and the effect on EphB2‐mediated cell repulsion in the Co115 co‐cultures was evaluated. Scale bar, 500 μm. *E*, Cell mixing was quantified by counting the percentage of EGFP‐positive cells forming clusters of various sizes in co‐cultures of EphB2‐ and ephrinB1‐expressing cells. In the control experiment a large proportion of cells were found in large (>30 cells) clusters, but this was dramatically reduced when clathrin‐mediated endocytosis was inhibited by either expression of dynamin1‐T65A or AP180 C‐terminus. Error bars represent standard deviation (*n* = 5 random fields per condition, ****P < 0.0001, Student's two‐tailed unpaired *t* test). *F,* Bar graph showing the quantification of EphB2‐mediated patterning of EphB2/ephrinB1 or control EphB2/RFP co‐cultures expressing control BFP, dynamin1‐T65A, or AP180 C‐terminus. Data represent mean ± standard error of the mean. ****P < 0.0001, Student's two‐tailed unpaired *t* test, at least 20 images were analysed in three independent experiments

To visualise EphB2‐mediated cell repulsion we used the colorectal cancer cell line Co115 stably expressing EphB2 and EGFP, ephrinB1 and RFP, EGFP alone and RFP alone.^5^ The cells were co‐cultured for 48 h, fixed, imaged and the size of the clusters of EGFP‐expressing cells was quantified as previously described.^5^ During the co‐culture the initially randomly mixed EphB2‐ and ephrinB1‐expressing cells repelled over time to minimise contact and formed homogenous clusters (groups) of cells expressing either EphB2 receptor or ephrinB1 ligand, resulting in a pattern formation of the two cell lines, which is distinct from the co‐culture of EphB2‐expressing cells with RFP or two cell lines that do not express EphB receptor or ligand (Figure 1A). Quantification of the percentage of clusters of EGFP‐positive cells showed that EphB2‐expressing cells formed large clusters when co‐cultured with ephrinB1‐expressing cells (Figure 1B), but were more mixed (a higher proportion of small EGFP‐positive cell clusters) when co‐cultured with control RFP‐expressing cells consistent with previous observations.^5^ Cell clusters containing >31 cells were not present in control co‐cultures (Figure 1B). As the ephrins are involved in tissue patterning, we applied an established method for pattern analysis on our EphB‐mediated cell repulsion assay that has been developed for analysing chimeric patterning in retina tissues.^25^, ^26^ This method has been shown to distinguish between random mixing and clustered patterns formed by two cell populations both in tissue and in computer models.^26^ It has the advantage of correcting for unequal proportions of the two populations that may arise at the seeding stage of the repulsion assay in the ‘pattern score’ that is generated.^25^ The images collected from our assay showed striking differences in patterning (Figure 1A). The results showed that the method reliably discriminates between cell clustering in EphB/ephrinB co‐culture and the random cell mixing in the two controls where RFP‐expressing cells were co‐cultured with either EphB2‐ or EGFP‐expressing Co115 cells (Figure 1C; *P* < 0.0001, Student's *t* test). Thus, we conclude that image analysis of patterning can be applied to EphB‐ephrinB cell repulsion studies as we found it to produce reproducible data in agreement with previously published findings.^5^

To analyse whether EphB2‐mediated cell repulsion was dependent on the GTPase dynamin‐1, a scission molecule that is involved in most endocytic pathways to sever membrane buds from the plasma membrane, we overexpressed the dominant negative GTPase mutant T65A in our co‐cultures.^27^ Inhibition of dynamin‐mediated membrane scission strongly reduced the clustering of EphB receptor and ephrinB1 expressing cells (Figure 1D‐F; *P* < 0.0001). Because multiple endocytic pathways are dependent on dynamin‐1 for membrane scission we next used a specific reagent to block clathrin‐mediated endocytosis, AP180 C‐terminus. AP180 is a membrane‐binding protein that promotes clathrin polymerisation and formation of endocytic membrane buds.^28^, ^29^ The C‐terminus of AP180 has multiple clathrin‐binding motifs and overexpression of this domain efficiently blocks internalisation of receptors that are internalised via clathrin‐mediated endocytosis.^29^, ^30^, ^31^ Expression of AP180 C‐terminus in the Co115 co‐cultures with EphB2 (GFP) and ephrinB1 (RFP) inhibited EphB/ephrinB cell patterning and increased cell mixing (Figure 1D, F; *P* < 0.0001), thus establishing that EphB2‐mediated cell repulsion is regulated by clathrin‐mediated endocytosis.

### 2.2 EphB2 interacts with Eps15R and Eps15 via Numb

**Figure 2:**
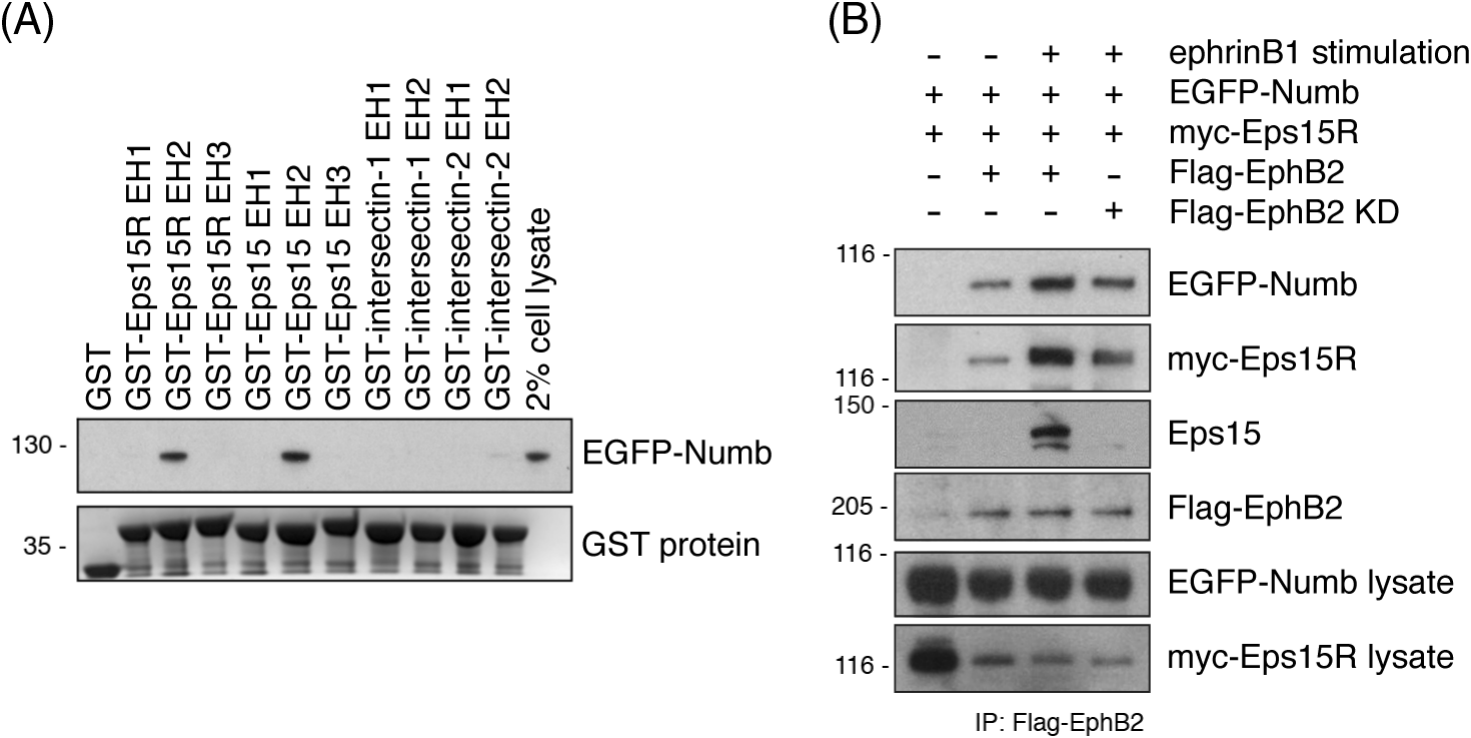
**EphB2 interacts with Numb and Eps15R.** *A,* A limited screen for Numb interaction with individual EH domains from Eps15R, Eps15, intersectin‐1, and intersectin‐2. GST‐tagged EH domains were used in a pull‐down assay with lysates from EGFP‐Numb expressing HEK293T cells and analysed by Western blot. A Coomassie stained SDS‐PAGE gels shows the equal loading of the EH domains. GST alone was used as a control. *B,* Co‐immunoprecipitation analysis from HEK293T cells expressing Flag‐EphB2, EGFP‐Numb, and myc‐Eps15R. A kinase dead (KD) EphB2 mutant was used as a control. The cells were stimulated with pre‐clustered soluble ephrinB1 ligand. A Flag antibody was used for immunoprecipitation and co‐immunoprecipitation was assessed by Western blot

To further our understanding of EphB2 trans‐endocytosis we next sought to identify novel components of the endocytic complex. Studies using immuno‐precipitation and mass spectrometry methods to map the interactome of EphB2 have not yielded data that identify endocytic proteins.^32^, ^33^ We therefore decided to target our search to the previously identified endocytic adaptor protein of EphB2, Numb.^18^ The interaction between Numb and EphB2 is mediated by the phosphotyrosine‐binding (PTB) domain of Numb, and is functionally important during neural development.^18^ Numb has previously been implicated in receptor‐mediated endocytosis, particularly of receptor tyrosine kinases.^19^ It is comprised of a PTB domain that binds to activated receptor tyrosine kinases, a motif domain containing proline‐rich motifs binding SH3 domains, a single DPF motif that binds the clathrin adaptor AP2, and two NPF motifs that bind EH‐domain containing proteins.^18^, ^22^, ^34^, ^35^ AP2 is the major adaptor protein for bringing in cargo to clathrin‐coated pits. However the weak affinity interaction of a single DPF motif binding to AP2 (K_d_ ≈ 1 μM) is not sufficient for formation of a stable protein complex that can nucleate clathrin coat formation.^24^, ^36^ We therefore performed a small interaction screen of EH domain‐containing endocytic proteins to identify interaction partners of the NPF motifs in Numb. The NPF motif binds EH domains and there are a number of endocytic scaffold proteins that contain multiple EH domains; Eps15, Eps15R, intersectin‐1, intersectin‐2. EH domain scaffold proteins serve important functions in clathrin‐mediated endocytosis, in particular by facilitating the formation of multi‐protein complexes.^37^, ^38^, ^39^ To investigate which endocytic EH‐domain containing proteins interacted with Numb, a screen was performed with EH domains of Eps15R, Eps15, intersectin‐1, and intersectin‐2 expressed as recombinant GST‐tagged protein and immobilised on beads. We found that Numb specifically bound the second EH domains of Eps15R and Eps15, but not the EH domains of intersectin‐1 or ‐2 (Figure 2A). Thus, Numb has a binding specificity for certain EH domains. We found that Eps15R and Eps15 both form a complex with EphB2 in vivo together with Numb, as shown in a co‐immunoprecipitation assay from HEK293T cells (Figure 2B). The interaction was more prominent upon stimulation of the EphB2‐expressing cells with pre‐clustered extracellular domain of ephrinB1. A kinase dead mutant of EphB2 was used as a control for ligand activation. We suggest that this protein complex consisting of EphB2‐Numb‐Eps15/R could function as the adaptor complex for EphB2 endocytosis mediated by clathrin, which has not been described previously. We therefore pursued further study of the role of Eps15R and Eps15 in the endocytosis of EphB2 receptor.

### 2.3 The role of Eps15R and Eps15 in clathrin‐mediated endocytosis

Having established that trans‐endocytosis is clathrin‐mediated and that the EphB2 receptor forms a complex with Eps15R and Eps15, we next wanted to explore the molecular mechanisms further. Eps15 and Eps15R have been suggested to not have a significant role in clathrin‐mediated endocytosis based on receptor uptake studies as only small inhibitory effects on EGFR and transferrin uptake are observed when it is knocked down.^37^, ^40^ However, acute perturbation of Eps15R interactions by microinjection of antibodies into cells shows a profound inhibition of endocytosis of EGF, suggesting that it has an important role in endocytosis of EGFR.^21^

**Figure 3:**
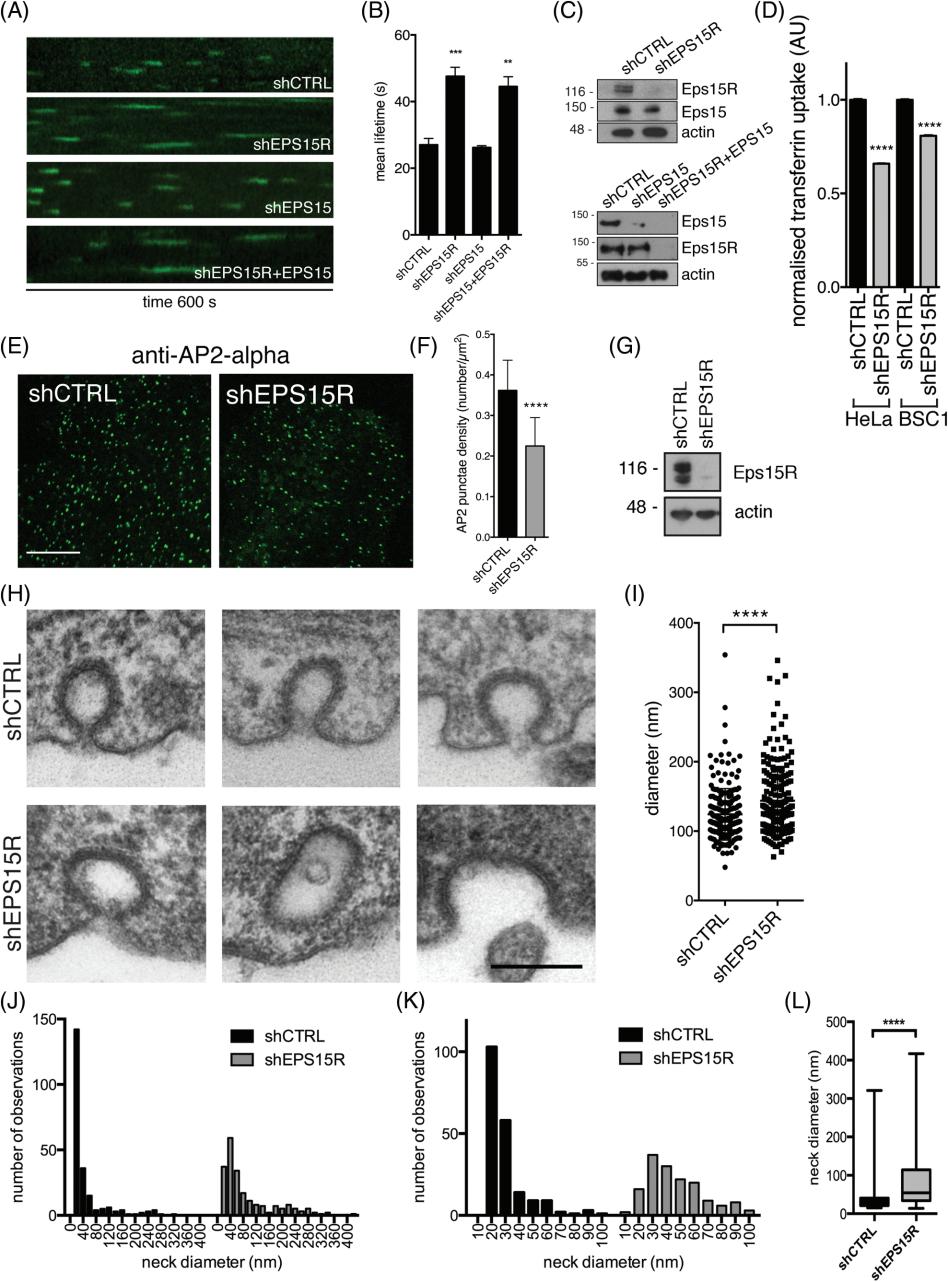
**Eps15R regulates clathrin‐mediated endocytosis.** *A*, Live cell imaging of BSC1 cells stably expressing AP2‐σ2‐EGFP. Kymographs (10 minutes) from cells treated with control shRNA, shEPS15R, shEPS15 or shEPS15R + shEPS15 prior to imaging are shown. *B*, Quantification of AP2‐σ2‐EGFP lifetime on the plasma membrane in BSC1 cells. ***P < 0.001, **P < 0.01 Student's two‐tailed unpaired test, *n* = 3 independent experiments. Mean ± standard error of mean. *C,* Western blot analysis of BSC1 cell lysates to assess Eps15R and Eps15 knockdown efficiency. *D,* Uptake of Alexa‐546‐transferrin in HeLa and BSC1 cells treated with control shRNA or shEPS15R, measured in a FACS analyser. ****P < 0.0001; Student's two‐tailed unpaired test; *n* = 30 000 (HeLa), *n* = 5000 (BSC1). Data were normalised to shCTRL cells and given as mean ± standard error of the mean. *E*, Endogenous AP2 staining in HeLa cells treated with control or EPS15R shRNA (shCTRL, shEPS15R). Scale bar 10 μm. *F*, Bar graph showing quantification of the density of AP2 punctae in control and Eps15R knockdown HeLa cells shown in D. ****P < 0.0001; two‐tailed unpaired Student's *t* test, *n* = 70. Data represent mean ± standard deviation. *G,* Western blot showing the level of knockdown of endogenous Eps15R in HeLa cell lysate. *H,* Diameter of clathrin‐coated structures (****P < 0.0001; two‐tailed unpaired Student's *t* test, *n* = 200). Mean ± standard deviation. *I,* Diameter of clathrin‐coated structures (****P < 0.0001; two‐tailed unpaired Student's *t* test, *n* = 200). Mean ± standard deviation. *J,* Histogram showing the distribution of CCP neck width, *n* = 200. *K,* Histogram showing the distribution of CCP neck from a subset of the data set with a neck width of 0‐100 nm. *L,* Quantification of the CCP neck width presented in a box and whiskers plot (****P < 0.0001; Mann‐Whitney test, *n* = 200)

To study the kinetics of clathrin‐coated pit formation we used live cell imaging of BSC1 cells stably expressing AP2‐σ2‐EGFP (Figure 3A‐B). These cells lend themselves to live cell imaging due to their large size and flat shape. Cells were treated with shRNA against Eps15R, Eps15 or Eps15R + Eps15 and the lifetime of AP2‐σ2‐EGFP was quantified (Figure 3A‐C). In Eps15R knockdown cells the AP2 punctae had a significantly longer lifetime (42 s) compared to cells treated with control shRNA (shCTRL, 27 s), reflecting a slowing of clathrin‐coated pit maturation (Figure 3B). Knockdown of Eps15 did not have a significant effect compared to control (26 s), and knockdown of both Eps15R and Eps15 did not show an additional increase in lifetime compared to knockdown of Eps15R alone (Figure 3A‐B). Western blot analysis of BSC1 cell lysates showed an efficient knockdown of Eps15R or Eps15 protein levels, and did not show a compensatory increase in either Eps15 or Eps15R expression (Figure 3C). This suggested that Eps15R and Eps15 are functionally diverse and we therefore focused further experiments on Eps15R.

Knockdown of Eps15R showed a reduced uptake of fluorescent transferrin in both HeLa and BSC1 cells treated with shRNA targeting the EPS15R gene (shEPS15R; Figure 3D) demonstrating that clathrin‐mediated endocytosis was significantly inhibited when Eps15R was depleted. We analysed a large number of cells by FACS (fluorescence activated cell sorting), which may explain why a significant difference in transferrin uptake was observed contrary to previous studies where smaller sample sizes have been analysed.^37^, ^40^ Furthermore, using confocal microscopy we observed a significant reduction in the density of clathrin‐coated pits (CCPs), on the plasma membrane of HeLa cells where Eps15R had been knocked down (Figure 3E‐G). To investigate whether the knockdown of Eps15R changed the size or shape of CCPs, high‐resolution images were collected using transmission electron microscopy. Morphometric analysis showed an increase in the mean diameter of CCPs and vesicles (Figure 3H, I; 124 nm versus 143 nm). In addition, an increase in the proportion of open versus closed CCPs was observed in the Eps15R deficient cells (42% versus 24%). A quantification of the width of the open neck of coated pits of all different stages of invagination was performed which provided a measure of clathrin‐coated vesicle closure. A significant increase in the average diameter of the neck of the clathrin‐coated membrane buds in Eps15R knockdown cells was observed, shown here in electron micrographs and histograms of the frequency distribution of neck diameters (Figure 3H, J). Focusing in on the diameter of necks of 10‐100 nm clearly illustrated the differences between control and Eps15R knockdown cells (Figure 3K). While control clathrin‐coated intermediates had a median neck diameter of 26 nm the intermediates from Eps15R deficient cells had a median neck diameter of 54 nm (Figure 3L). A similar phenotype was previously observed when proteins that regulate clathrin assembly; amphiphysin‐1, CALM or NECAP‐1, were knocked down or functionally perturbed.^41^, ^42^, ^43^, ^44^ The phenotype that we describe here for Eps15R depletion; slowing of CCP maturation, reduced transferrin receptor uptake, and aberrant clathrin coat formation; thus point towards a functional role for Eps15R in regulation of clathrin coat assembly.

### 2.4 Eps15R formed a direct interaction with clathrin heavy chain terminal domain

**Figure 4:**
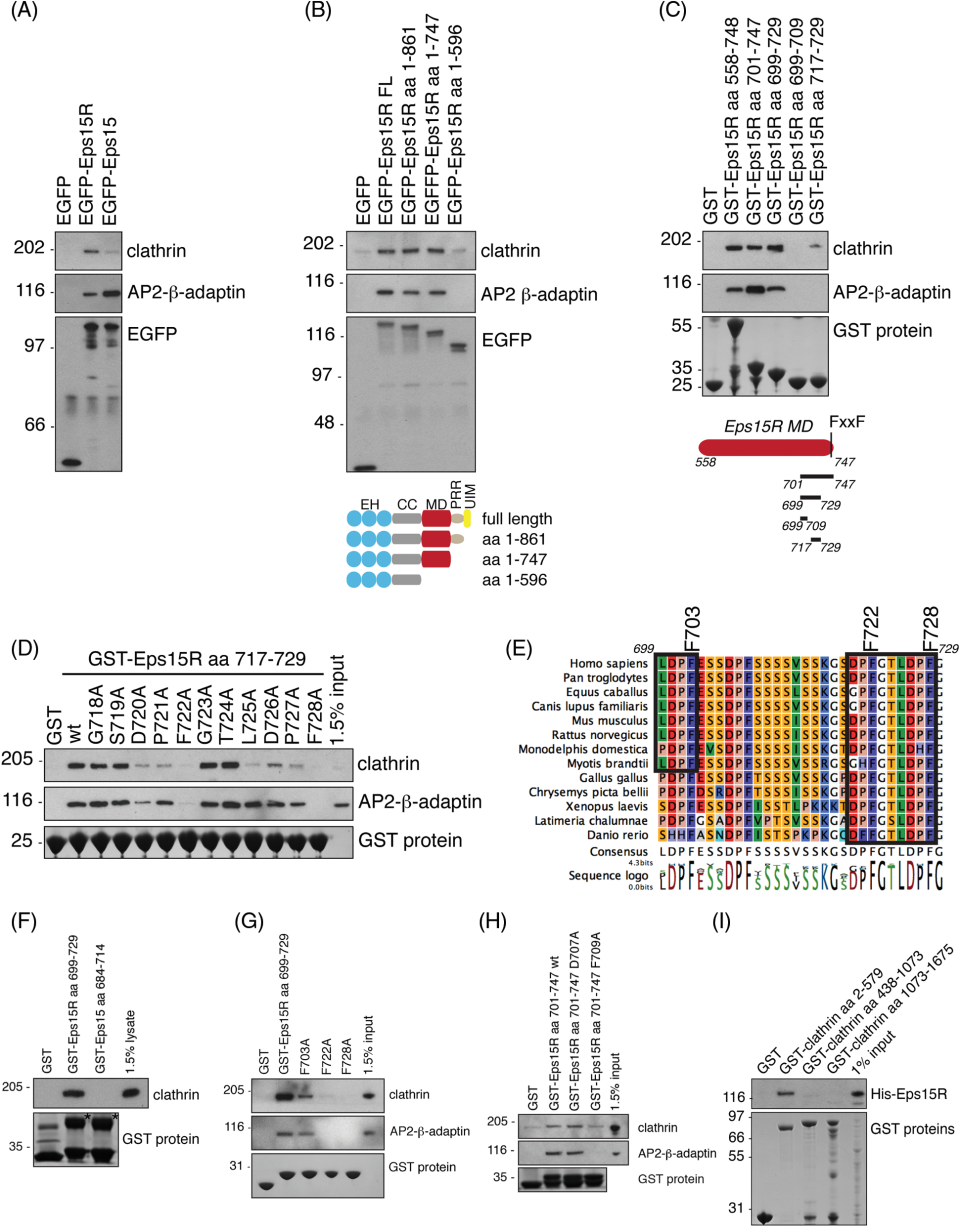
**Eps15R interacts directly with clathrin heavy chain terminal domain.** *A,* The interaction between Eps15R and clathrin were analysed in a co‐immunoprecipitation assay where EGFP‐Eps15R was immunoprecipitated from HeLa cell extract. EGFP and EGFP‐Eps15 were used as controls. The samples were immunoblotted for clathrin, AP2 β‐adaptin, and EGFP. *B*, Immunoprecipitation of C‐terminally truncated EGFP‐Eps15R constructs expressed in HeLa cells identified the motif domain (aa 596‐747) as the clathrin‐binding region. Deletion of the UIM domains (aa 1‐861) or the proline‐rich region (aa 1‐747) did not affect clathrin binding. The samples were analysed by immunoblotting for clathrin, AP2‐β‐adaptin, and EGFP. *C,* The interaction between clathrin and the Eps15R motif domain (aa 558‐747) was analysed by GST pull‐downs. GST‐tagged deletion constructs of the motif domain showed that the minimal peptide that bound clathrin was aa 717‐729. *D,* GST pull‐down assay using alanine scanning mutations of the Eps15R clathrin‐binding region (aa 717‐729) identified a novel clathrin‐binding motif, DPFxxLDPF. GST‐fusion proteins were incubated with detergent extracts from HeLa cells and immunoblotted with antibodies against clathrin and AP2‐β‐adaptin. *E,* Sequence alignment of Eps15R aa 699–729 (mouse) from different species showed that the DPFxxLDPF (aa 720‐728) motif is conserved in vertebrates. The sequence annotation refers to the mouse protein sequence. In addition, it highlighted an additional LDPF motif (L700) that was conserved in mammals. Chimpanzee (Pan troglodytes), horse (Equus caballus), dog (Canis lupus familiaris), mouse (*Mus muscularis*), rat (Rattus norvegicus), opossum (Monodelphis domestica), bat (*Myotis brandtii*), chicken (Gallus gallus), turtle (Chrysemys picta bellii), frog (Xenopus laevis), coelacanth (*Latimera chalumnae*), zebrafish (Danio rerio). *F,* The clathrin binding to Eps15R (aa 699‐729) and Eps15 (aa 687‐723) motif domains was compared in a GST pull‐down assay. The asterisks point to the GST‐tagged motif domain, and the band below in the lane is GST alone. *G,* Site‐directed mutagenesis of amino acids F703, F722, F723 reduced the binding of clathrin to GST‐Eps15R aa 699‐729 from HeLa lysate in a GST pull‐down assay. Samples were analysed by immunoblotting for clathrin and AP2. *H,* GST pull‐down using Eps15R aa 701‐747 and mutants of a DPF motif. Samples were analysed by immunoblotting for clathrin and AP2 *I,* The interactions between GST‐clathrin heavy chain and recombinant full‐length Eps15R were analysed in a GST pull‐down assay. GST fusion proteins comprising the clathrin terminal domain (aa 2‐579), distal domain (aa 438‐1073), and proximal domain (aa 1073‐1675) were incubated with detergent extracts of Sf9 cells expressing full‐length His‐Eps15R. Samples were analysed by immunoblotting for His‐tagged Eps15R

The prominent effect on clathrin‐coat morphology following Eps15R knockdown led us to next examine whether Eps15R interacted directly with clathrin. Clathrin showed a stronger association with EGFP‐Eps15R compared to EGFP‐Eps15 in a co‐immunoprecipitation assay from HeLa cell extracts (Figure 4A). No clathrin was co‐immunoprecipitated by control EGFP. AP2 was used as a control and was co‐immunoprecipitated by both EGFP‐Eps15R and EGFP‐Eps15. Thus, Eps15R can bind both clathrin and AP2 in vivo. We next mapped the region responsible for clathrin‐binding using C‐terminally truncated constructs of EGFP‐Eps15R. Deletion of the ubiquitin interacting motif (UIM) domains (leaving aa 1‐861) or UIMs plus the proline‐rich region (leaving aa 1‐747) did not impact on clathrin co‐immunoprecipitation, whereas a construct (aa 1‐596) additionally lacking the motif domain lost both clathrin and AP2 binding (Figure 4B). To further narrow down the clathrin‐binding site we used GST‐tagged constructs comprising the motif domain and truncations thereof in a pull‐down assay. The shortest construct that bound clathrin from HeLa cell extract was aa 717‐729 (Figure 4C). However, it should be noted that a slightly longer construct (aa 699‐729) bound clathrin more efficiently (Figure 4C). To identify a clathrin‐binding motif in Eps15R, we used the shortest construct identified in Figure 4C and mutated individual amino acids (aa 718‐728) to alanine residues. These mutated peptides were used in a GST pull‐down assay. Our affinity purification assay using multiple peptides with alanine substitutions identified a binding motif between Eps15R and clathrin, DPFxxLDPF (Figure 4D). An alignment of Eps15R orthologues showed that the DPFxxLDPF motif was conserved across vertebrates, and highlighted an additional LDPF motif starting at amino acid 700 that is only present in placental mammals (Figure 4E, Figure S1, Supporting Information). In comparison, Eps15 does not contain any LDPF motifs. Pair‐wise sequence alignment of Eps15R aa 699‐729 with the corresponding DPF‐containing region of Eps15 (aa 687‐723) only showed a 14.6% identity. We compared the binding of clathrin to this region of Eps15R and Eps15 in a GST pull‐down, and found that Eps15R but not Eps15 associated with clathrin (Figure 4F). Next we investigated the significance of having two LDPF motifs in tandem in the Eps15R sequence. The first LDPF sequence (aa 699‐709) did not show significant binding to clathrin on its own (Figure 4C). We investigated whether this additional LDPF motif could explain the increased binding efficiency of Eps15R aa 699‐729 compared to aa 717‐729 (Figure 4C). Indeed, mutation of F703A, F722A, or F728A in a longer Eps15R peptide (aa 699‐729) reduced clathrin binding (Figure 4G). The LDPF motif is a non‐canonical clathrin motif, similar to the classic clathrin boxes DLL, LLxLD and PWDxW that contain hydrophobic residues and interact with clathrin heavy chain terminal domain.^45^, ^46^, ^47^, ^48^ Finally, to investigate whether the DPF motif that is conserved between the two LDPF motifs would also contribute to clathrin binding we compared two mutants, D707A and F709A from this DPF motif, to wild type Eps15R and found that F709A but not D707A reduced clathrin binding (Figure 4H). In summary, having two LDPF motifs in tandem in Eps15R increased the binding efficiency of clathrin and the addition of a DPF motif 2 amino acids upstream of a LDPF motif also increased the clathrin association with Eps15R. To investigate which region of clathrin heavy chain Eps15R interacted with, we performed a pull‐down assay with purified domains of clathrin and recombinant full‐length Eps15R. Only the N‐terminal domain of clathrin bound Eps15R efficiently (Figure 4I). We concluded that Eps15R associated directly with the clathrin terminal domain via a non‐canonical motif, DPFxxLDPF.

### 2.5 Overexpression of single Eps15R mutants F703, F722, F728 in the clathrin‐binding motif are not critical for the subcellular localisation of clathrin

To investigate whether the DPFxxLDPF motif in Eps15R directs the localisation of clathrin, we transiently overexpressed Eps15R containing a wild type clathrin‐motif or alanine mutants of residues 703, 722 or 728. The co‐localisation with clathrin in HeLa cells was examined using confocal microscopy. All three single mutants showed a punctate pattern that co‐localised with clathrin at the plasma membrane (Figure S2A‐D). Quantification of the size of the punctae did not show a significant difference to wild type Eps15R (Figure S2E). These results suggest that in the context of full‐length Eps15R individual mutations in the identified clathrin‐binding motif do not reduce the affinity for clathrin enough to affect recruitment of clathrin to the plasma membrane, and we therefore pursued experiments using a triple alanine mutation comprising the three phenylalanines, F703A/F722A/F728A.

### 2.6 Eps15R regulates AP2‐clathrin complex formation in vitro and in vivo

**Figure 5:**
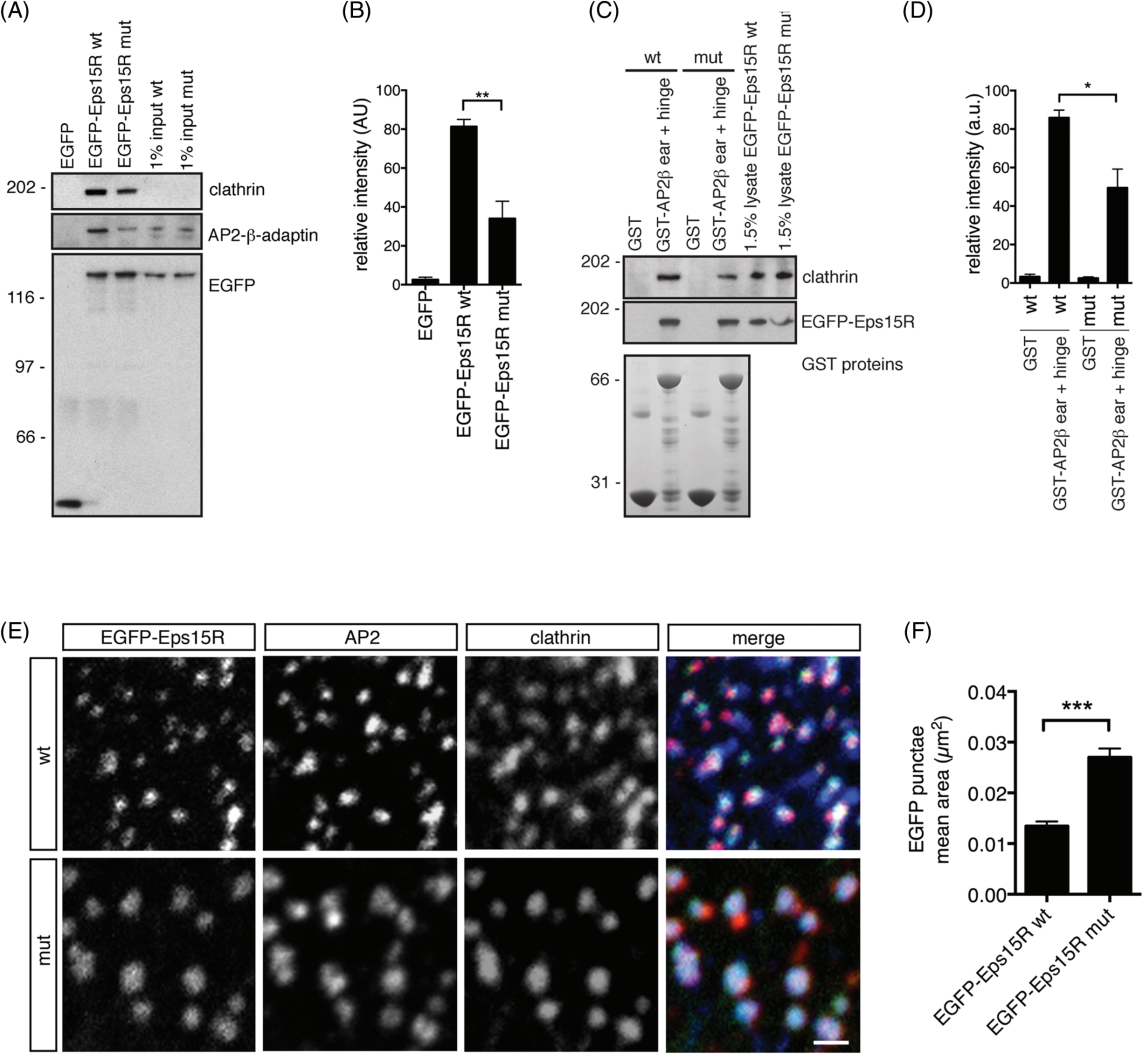
**Eps15R regulates AP2‐clathrin complex formation in vitro and in vivo.** *A,* Immuoprecipitates from HeLa lysates of EGFP‐Eps15R wild type (wt) or mutant (mut; F703A/F722A/F728A) were analysed by immunoblotting for clathrin, AP2 β‐adaptin, and EGFP to ensure equal loading. *B,* Quantification of Western blots from three independent experiments described in A. Mean ± standard error of the mean. **P < 0.01, Student's two‐tailed unpaired *t* test. *C*, A GST pull‐down assay using AP2‐β‐appendage with hinge, a construct that binds clathrin (aa 616‐937^36^;). Lysates from HeLa cells expressing EGFP‐Eps15R wild type (wt) or clathrin‐binding mutant (F703A, F722A, F728A) were used. Samples were analysed with clathrin and EGFP immunoblotting. *D,* Bar graph showing the quantification of three independent experiments described in C. Data represent mean ± standard error of the mean. *P < 0.05, Student's two‐tailed unpaired *t* test. *E*, Immunofluorescence images of wild type EGFP‐Eps15R, mutant EGFP‐Eps15R (F703A, F722A, F728A) and endogenous AP2 and clathrin stain in HeLa cells. Scale bar 1 μm. *F,* Bar graph showing the quantification of wild type and mutant EGFP‐Eps15R fluorescent punctae (shown in E, G) in five independent experiments. Data represent mean ± standard error of the mean. ***P < 0.0001, Student's two‐tailed unpaired test, *n* = 50

To investigate whether the Eps15R F703A/F722A/F728A triple mutant (Eps15R mut) associated less readily with clathrin we performed a co‐immmunoprecipitaiton assay. Immunoprecipitation of the mutant Eps15R (EGFP‐Eps15Rmut) from cell lysates showed that less clathrin was co‐precipitated compared to the wild‐type protein (Figure 5A). Quantification of the clathrin immunoblots from three independent experiments showed a halving of the amount of clathrin that was co‐immunoprecipitated with Eps15Rmut compared to wild type protein (Figure 5B). The amount of AP2 that co‐immunoprecipitated with Eps15Rmut was also reduced, and we hypothesise that it was likely to be due to the reduced amount of clathrin that was accumulated since the high‐affinity AP2‐binding site (FxxF) in Eps15R was not compromised. To examine whether Eps15R may strengthen clathrin‐AP2 interactions by providing an expanded binding platform for these proteins, we examined the effect on clathrin‐binding to AP2‐β2‐appendage with its hinge (aa 616‐937) in lysates from cells over‐expressing wild type and mutant Eps15R (Figure 5C). AP2 functions as an important adaptor for clathrin and it contains clathrin‐binding motifs in its β2 hinge, and is known to be a key factor in regulating clathrin polymerisation.^23^, ^36^, ^49^ To investigate the contribution of Eps15R to the clathrin‐AP2 interaction and how efficiently the AP2‐clathrin complex forms in the presence or absence of Eps15R we performed GST pull‐downs with AP2 β2‐hinge containing the clathrin‐binding motif. Western blot analysis of the pull‐down experiments indicated that a strong reduction of clathrin association with AP2‐β2‐appendage‐hinge occurred in samples where mutant Eps15R was over‐expressed (Figure 5C‐D). Similar amounts of wild type and mutant Eps15R were bound to AP2‐β2‐appendage‐hinge and the expression of clathrin in the two cell extracts was similar (Figure 5C, D). Thus, these experiments suggest that Eps15R regulates clathrin‐AP2 interactions. The clathrin‐AP2 interaction has been suggested to be the dominant mechanism that controls clathrin coat assembly,^23^ and we hypothesise that Eps15R adds an additional layer of regulation to this mechanism.

Expression of the triple mutant EGFP‐Eps15R (F703, F722, F728) in HeLa cells showed that it still targeted to the plasma membrane and co‐localised with clathrin and AP2 (Figure 5E‐F). Thus, mutation of the LFPF motifs did not perturb the trafficking of Eps15R, and suggested that Eps15R is not recruited to clathrin‐coated pits by clathrin. This is not surprising based on the literature available describing the high avidity protein interactions for Eps15, AP2 and clathrin and furthermore the potential for heterodimerization between Eps15R and Eps15.^23^, ^36^ However, we did notice that our mutant Eps15R formed expanded punctae compared to wild type Eps15R (Figure 5E‐G), implying that it does have an impact on clathrin coat formation. Based on our biochemistry data we suggest that this is due to regulation of clathrin‐AP2 interactions. Taken together these results suggest that Eps15R is not directly recruited by clathrin, but can regulate the maturation of clathrin‐coated pits together with clathrin adaptor proteins such as AP2.

### 2.7 The Eps15R‐clathrin interaction is necessary for EphB2‐mediated cell‐cell repulsion

**Figure 6:**
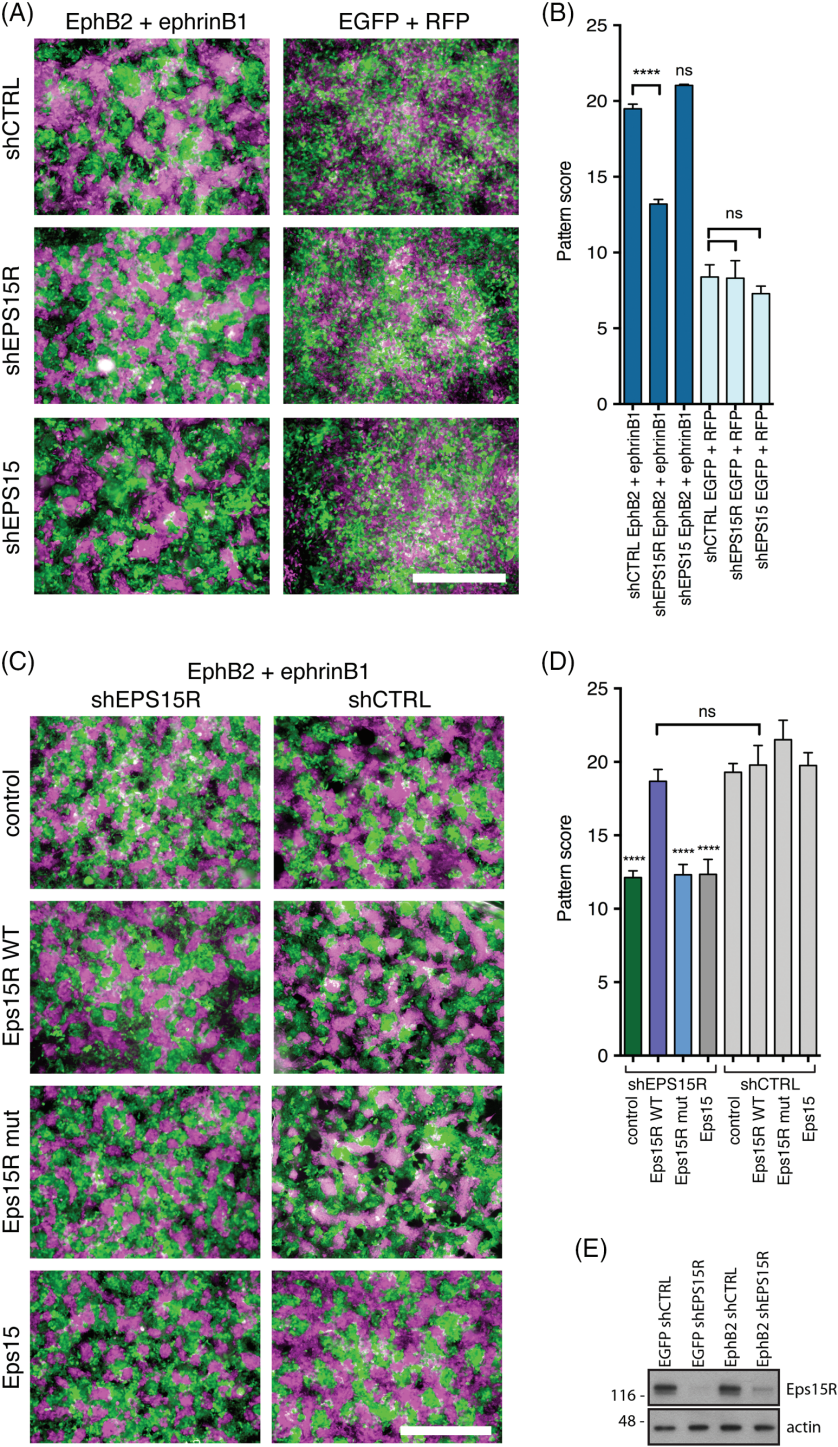
**The role of Eps15R in EphB2 trans‐endocytosis.** *A,* Co‐culture of Co115 cells stably expressing EphB2 (EGFP) or ephrinB1 (RFP). RFP has been pseudocoloured in magenta for visualisation purposes. Cells expressing EphB2 (EGFP) were treated with shRNA prior to co‐culture. Scale bar, 500 μm. *B,* Bar graph showing the mean pattern score for EphB2/ephrinB1 co‐cultures (dark blue). Control EGFP/RFP co‐cultures of Co115 cells that do not express EphB receptor and ligand show random mixing of cells (light blue). Data represent mean ± standard error of the mean. ****P < 0.0001, Student's two‐tailed unpaired *t* test, *n* > 20 in four independent experiments. *C,* Epifluorescence images of EphB2‐ephrinB1 co‐cultures where EphB2‐expressing cells were treated with either shEPS15R or control shRNA. To rescue of the patterning defect as a result of Eps15R knockdown myc‐Eps15R, myc‐Eps15R mutant (F703A, F722A, F728A), myc‐Eps15 or control BFP was expressed. Scale bar, 500 μm. *D,* Quantification of patterning as a result of cell repulsion in co‐cultures of Co115 cells expressing EphB2 and ephrinB1. Data represent mean ± standard error of the mean. ****P < 0.0001, Student's two‐tailed unpaired *t* test, *n* > 20 in four independent experiments

Finally, we wanted to examine the significance of the Eps15R‐clathrin interaction in trans‐endocytosis of EphB/ephrinB complexes. We again used the Co115 co‐culture assay. First, we stably knocked down Eps15R in EphB2‐expressing cells and co‐cultured them with ephrinB1‐expressing cells or control RFP‐expressing cells (Figure 6A‐B). We quantified the cell pattern score as before, and showed that treatment with Eps15R shRNA resulted in a significant reduction in EphB2‐mediated patterning (Figure 6A, B). However, it should be noted that we did not observe a complete inhibition of cell‐cell repulsion as the cells are not mixed to the same extent as the controls (Figure 6A, B). These observations are in agreement with the slowing rather than complete inhibition of clathrin‐mediated endocytosis that we demonstrated earlier by live cell imaging of CCPs (Figure 3A, B). Knockdown of Eps15 did not significantly alter the patterning compared to the control (Figure 6A, B).

Finally, we performed a rescue experiment to restore trans‐endocytosis of the EphB2‐ephrinB1 complex, and cell‐cell repulsion, in Eps15R knockdown cells (shEPS15R) by re‐expression of wild type or mutant Eps15R (F703A/F722A/F728A). The trans‐endocytosis, and hence the repulsion and EphB2‐mediated patterning, was restored to normal by expression of wild type Eps15R but not by the clathrin‐binding mutant Eps15Rmut (Figure 6C, D), demonstrating the functional importance of the Eps15R‐clathrin interaction. As a control, we overexpressed full‐length Eps15 and showed that it cannot compensate for the loss of Eps15R (Figure 6C, D). Importantly, this demonstrated that Eps15R has a distinct function that is not redundant in Eps15. Taken together these results show that the interaction between Eps15R and clathrin is critically important to maintain functional endocytosis of the EphB2‐ephrinB1 complex resulting in cell‐cell repulsion in cell culture.

## 3 DISCUSSION

Contact‐mediated cell repulsion mediated by EphB‐ephrinB interactions is important during development, synaptic plasticity, and in cancer progression.^1^, ^50^, ^51^ In order to understand how the EphB‐ephrinB complex is trans‐endocytosed, identification of novel interactions with endocytic proteins that are bound to the complex is necessary, as this is a poorly defined mechanism. Studies using proteomics approaches to identify novel interaction partners of the EphB2 receptor have not yet identified proteins involved in receptor trafficking.^32^, ^33^ In this study we therefore instead used a targeted approach and employed the EphB2 interaction partner Numb^18^ as a starting point to show that it directly interacted with endocytic proteins Eps15 and Eps15R. This protein complex, EphB2/Numb/Eps15/Eps15R, formed upon receptor‐stimulation with soluble ephrinB1 ligand. Here we report the interaction of endocytic protein Eps15R with the EphB2 receptor and how its interaction with clathrin was required to facilitate trans‐endocytosis. Specific perturbation of clathrin‐mediated endocytosis by overexpressing AP180 C‐terminus provided the first evidence that EphB2 trans‐endocytosis is clathrin‐dependent. Knocking down expression of Eps15R, but not Eps15, reduced EphB2‐mediated cell repulsion and thus demonstrated the significance of this endocytic accessory protein in trans‐endocytosis and furthermore showed that Eps15R and Eps15 are not functionally redundant in this context.

Clathrin‐mediated and non‐clathrin‐mediated endocytosis pathways are important for internalisation of different cargos under different conditions.^52^, ^53^ Trans‐endocytosis of EphB receptors and ephrinB ligands is known to be actin dependent.^10^, ^11^, ^16^ In general, the actin cytoskeleton is involved in internalisation of larger endocytic intermediates such as macropinocytosis, phagocytosis and bacterial internalisation but is generally not associated with clathrin‐mediated endocytosis.^53^, ^54^ Because the EphB/ephrinB endocytic membrane invaginations are large assemblies containing plasma membranes from two neighbouring cells the requirement of force generated by the actin cytoskeleton could be compared to cells under high membrane tension or with a polarised membrane where clathrin‐mediated receptor endocytosis is actin‐dependent.^55^, ^56^ It was recently shown that the mechanism of internalisation for soluble ephrinB1 ligand is different to that of membrane bound ligand,^16^ which may explain the discrepancy between our results and data from other groups suggesting that trans‐endocytosis is actin‐dependent but clathrin‐independent.^10^, ^11^ In our experiments we used co‐cultures of cells expressing full‐length EphB2 and ephrinB1. Moreover previous studies only assessed co‐localisation of clathrin with EphB and ephrinB rather than perturbing clathrin interactions. We suggest that EphB trans‐endocytosis is both actin‐ and clathrin‐dependent.

We identified a novel interaction between Eps15R and clathrin and showed that it is functionally important for EphB2 trans‐endocytosis in our rescue experiments. This interaction is mediated by a non‐canonical clathrin‐binding motif that binds to the terminal domain of clathrin heavy chain. We showed that this motif, DPFxxLDPF, binds better when arranged in tandem with an additional LDPF motif. Eps15R is the only human protein with two LDPF motifs in tandem, but there are a number of proteins that contain a PF‐(x)_n_‐LDPF motif. One example is BMP2K, a kinase that associates with clathrin‐coated vesicles and co‐immunoprecipitates with Numb but as of yet does not have a defined function in clathrin‐mediated endocytosis.^35^, ^57^ The data presented in this paper suggests a potential direct association between BMP2K and clathrin via this non‐canonical clathrin motif described in this paper. Peptides containing DPF motifs have been previously shown to bind to the clathrin terminal domain and AP2 with dual specificity.^58^ The ^725^LDPF in Eps15R is slightly different in that when mutated it loses clathrin binding more readily than AP2 association, which is an interesting observation. It is established that the spacing of DPF motifs is critical for determining binding specificity for both FCHo2 and AP2.^24^, ^59^ How this motif spacing is deciphered and decoded by the interaction partner is not known. In light of these studies, the differences between Eps15 versus Eps15R in terms of number of DPF motifs (13 compared to 16), the overall low amino acid conservation of the motif domains (25% identity) and the difference in spacing of the DPF motifs are likely to reflect functional differences. Here we show that clathrin associates more strongly with Eps15R compared to Eps15 and that the two clathrin‐binding LDPF motifs identified in Eps15R are not present in Eps15. Future research on the functional role of motif spacing in endocytic proteins will be important to further our understanding of the process.

Our study uncovers a novel link between Eps15R and clathrin that is functionally important. We hypothesise that Eps15R via its binding to clathrin may regulate clathrin coat assembly through formation of a stronger clathrin‐AP2 complex and a more stable coat, which is supported by our observation that knockdown of Eps15R increases pit size and reduces pit number. In our morphological analysis of Eps15R knockdown cells we discovered that Eps15R regulates the shape and size of clathrin‐coated pits, with a distinct increase in the width of the neck of the CCPs. Similar morphological phenotypes have been reported previously when clathrin‐binding proteins NECAP1 and CALM have been depleted.^42^, ^44^ The mechanism for how Eps15R would regulate clathrin assembly is not clear from our data, and would require further experiments. However, it is feasible that Eps15R acts as a molecular brace for clathrin at the rim of clathrin‐coated pits. Eps15R and Eps15 have a central coiled‐coil region that mediates anti‐parallel dimerization,^60^ which would position the clathrin motifs on opposite sides of an 18 nm rigid coiled‐coil. The distance between the vertices where the clathrin terminal domains are located in a spherical clathrin coat is 18.6 nm^61^. Eps15R could thus cross‐link terminal domains at a distance that would favour formation of a spherical clathrin cage.

In summary, we report that Eps15R and clathrin regulate EphB2‐mediated cell repulsion through trans‐endocytosis. Future studies are required to identify additional components involved in EphB2‐mediated trans‐endocytosis and the role of the clathrin and clathrin‐adaptor proteins such as Eps15R in cellular processes that are EphB‐mediated during development and in disease.

## 4 MATERIALS AND METHODS

### 4.1 Antibodies

Antibodies used in this study for immunofluorescence staining include: clathrin heavy chain mouse monoclonal (clone X22, 1:250 dilution), AP2 α‐adaptin mouse monoclonal (clone AP6, AbCam ab2730, 1:200 dilution), clathrin heavy chain rabbit polyclonal (AbCam, ab21679, 1:1500 dilution), Eps15R rabbit polyclonal (AbCam, ab53006, 1:200 dilution), FCHo2 rabbit polyclonal (Ra103; aa 525‐890, 1:200 dilution^31^), Eps15 rabbit polyclonal (Ra15, aa 530‐791; 1:500 dilution^62^). Goat anti‐mouse and anti‐rabbit Alexa‐488, ‐546 and ‐647 conjugated secondary antibodies were used at 1:500 dilution (Life Technologies). For immunoblotting the following antibodies were used: clathrin heavy chain (BD Transduction Laboratories, #610500, 1:10 000 dilution), AP2‐β‐adaptin (clone 100/1, Sigma, A4450, 1:5000 dilution), EGFP (AbCam, ab290, 1:20 000 dilution), Flag (clone M2, Sigma, F3165, 1:2000 dilution), His (GE Healthcare, #27‐4710‐01, 1:1000 dilution), β‐actin (AbCam, ab6276, 1:10 000 dilution), Eps15 (Santa Cruz, C‐20, 1:2000 dilution).

### 4.2 Plasmids

Rat AP2‐β appendage + hinge (aa 616‐937) was cloned into pGEX4T2. Mouse Eps15R motif domain constructs and mutants thereof were cloned in pGEX6p3 as follows: aa 558‐748; aa 701‐747; aa 699‐729; aa 699‐709; aa 717‐729. Human Eps15 motif domain aa 687‐723 was cloned in pGEX6p3. Human clathrin heavy chain domains; aa 2‐579, aa 438‐1073, aa 1073‐1675; were cloned in pGEX6p2. EH domains were cloned in pGEX6p3; Eps15 EH1 (aa 9‐103), Eps15 EH2 (aa 121‐215), Eps15 EH3 (aa 217‐313), intersectin‐1 EH1 (aa 14‐108), intersectin‐1 EH2 (aa 214‐309), intersectin‐2 EH1 (aa 14‐108), intersectin‐2 EH2 (aa 236‐331), Eps15R EH1 (aa 8‐104), Eps15R EH2 (aa 254‐362), Eps15R EH3 (aa 381‐564). C‐terminally truncated mouse Eps15R cloned in pEGFP‐C1 was kindly provided by Dr. A. Benmerah; aa 1‐907 (full‐length), aa 1‐861 (ΔUIM), aa 1‐747 (Δ proline‐rich region), aa 1‐596 (Δ motif domain). Full‐length human Eps15 was cloned in pCi‐N‐EGFP or pCi‐N‐myc. Full‐length human Numb was cloned in pEGFP‐C3. Full‐length shRNA‐resistant mouse Eps15R was cloned in pCi‐N‐EGFP or pCi‐N‐myc. Control shRNA from Sigma MISSION (CAACAAGATGAAGAGCACCAA), shEPS15 (GTTTGGGAGTTGAGTGATA) and shEPS15R (GTAAAGGGTTCTTGGACAA) were cloned into the pLKO1‐puro vector. Bovine dynamin1‐T65A was cloned in pCi‐N‐TagBFP and rat AP180‐C‐terminus (aa 530‐915) was cloned with a N‐terminal tag in pTagBFP‐C3.

### 4.3 Protein expression and purification

GST‐tagged proteins were expressed in *E. coli* BL21 cultured in terrific broth media at 37°C for 2 h following induction with 0.15 mM IPTG at OD_600_ 0.5‐0.7. The bacteria were harvested and lysed with a high‐pressure homogeniser (Constant Systems) in 50 mM HEPES pH 7.4, 500 mM NaCl, 2 mM DTT. The lysate was cleared by centrifugation in a Beckman Ti45 rotor at 30000 rpm for 25 min at 4°C. The supernatant was incubated with glutathione sepharose (GE Healthcare) for 10 min, spun down, washed extensively, and the beads were finally resuspended in 20 mM HEPES pH 7.4, 150 mM NaCl and 0.5 mM TCEP and used in pull‐down experiments.

### 4.4 Immunoprecipitations and GST pull‐downs

HeLa cells were homogenised in a lysis buffer containing: 20 mM HEPES pH 7.4, 100 mM NaCl, 0.5 mM TCEP, 0.1% (w/v) NP‐40, protease inhibitor cocktail set III (Calbiochem). For immunoprecipitates antibodies were added to the cleared lysate, incubated on a rotating wheel for 3.5 h at 4°C, protein A sepharose was added and the samples incubated for a further 0.5 h before they were washed extensively with the lysis buffer and eluted with sample buffer. The immunoprecipitates were analysed by immunoblotting. EphB2‐expressing cells were stimulated with 1 μg/mL pre‐clustered ephrinB1‐Fc (R&D Systems) for 20 min at 37°C prior to lysis and immunoprecipitation. For GST pull‐downs 100‐200 μg of lysate was added to GST‐tagged protein immobilised on beads and incubated for 30‐60 min at 4°C on a rotating wheel. The beads were pelleted and washed with lysis buffer multiple times. Bound protein was analysed by SDS‐PAGE and immunoblotting.

### 4.5 Cell Culture

HeLa, BSC1 σ2‐EGFP, and Co115 cells were cultured at 37°C and 5% CO_2_ in Dulbecco's modified Eagle's medium (DMEM) supplemented with 10% (v/v) foetal bovine serum. HeLa cells were purchased from the European Collection of Cell Cultures. BSC1 σ2‐EGFP cells^63^ were kindly provided by Dr. Kirchhausen (Harvard Medical School, USA). Co115 EGFP, Co115 RFP, Co115 EGFP:EphB2, and Co115 RFP:ephrinB1 cells^5^ were kindly provided by Dr. Batlle (Institute for Research in Biomedicine, Barcelona, Spain). All cell lines were tested for mycoplasma. Co115 cell lines in co‐cultures were plated at a density of 1.5‐2.0 × 10^5^ cells per cover slip and cultured for 2‐3 days in DMEM supplemented with 10% foetal bovine serum. In overexpression experiments cells were transfected with 1 μg/mL polyethylenimine (Sigma, #408727) 4‐8 hours after plating when cells were at 50‐80% confluency. For knockdown experiments the pLKO1‐puro vector was used to generate lentivirus, which was then applied to the cell cultures. After 48 h the cell lines were treated with puromycin for selection (HeLa 5 μg/mL, BSC1 σ2‐EGFP 10 μg/mL, Co115 1 μg/mL). The knockdown was confirmed by immunoblotting.

### 4.6 Epifluorescence, confocal microscopy and live cell imaging

Co‐cultures of Co115 cells were imaged on a Leica DM5500B epifluorescence microscope with a 10×/0.30 HCX PL Fluostar objective. Fixed samples were imaged on a Zeiss 780 confocal microscope equipped with a 63× (1.4NA) and a 10× objective. Images within an experiment were collected using fixed laser settings and exposure times. Images were analysed using ImageJ software (National Institutes of Health), and the ClonalTools macro was used for pattern analysis as described previously.^25^, ^26^ For pattern analysis each image was analysed by randomly applying perpendicular lines to the image and the corrected patch width along the lines was quantified. The analysis of percentage of EGFP‐positive cells was performed as in Cortina et al.^5^ In brief, the number of EGFP‐expressing cells growing in defined groups without mixing with RFP‐expressing cells were quantified in randomly selected areas of images and expressed as percentage of the total number of cells in that area. Clustered cells were defined as the number of cells of one population localised together without the disruption of cells from the second population, here RFP‐expressing cells.

Live cells, BSC1 AP2‐σ‐EGFP,^63^ were imaged at 37°C and 5% CO_2_ in a Perkin Elmer spinning disc microscope equipped with a 60× objective (Plan Apochromat VC, 1.4 NA, Nikon). Time‐lapse movies were collected at 2 s intervals for 10 min with a cooled EMCCD camera (9100/02, Hamamatsu). Quantitation of EGFP‐AP2 lifetimes was performed with Volocity software by generating kymographs. Randomly selected events were used in the analysis. In the case where the fluorescent punctae existed in both the first and last frame it was included in the analysis, otherwise only events that appeared and disappeared within the movie were analysed. A minimum of 700 events were analysed per group.

### 4.7 Transmission electron microscopy

HeLa cells were pelleted and fixed in 2.5% (v/v) glutaraldehyde, 2% (v/v) paraformaldehyde, 2% (w/v) tannic acid in phosphate buffered saline (pH 7.4), and then post‐fixed in 1% osmiumtetraoxide in 0.1 M cacaodylic acid (pH 7.4). The samples were dehydrated in a graded series of ethanol during which they were stained *en bloc* with 1% uranyl acetate in 70% ethanol, and then embedded in Durcupan resin (Fluka). Serial ultrathin sections (70 nm) were cut with a diamond knife (Diatome) on a Leica UltraCutE ultratome. The sections were collected on formvar‐coated copper grids, stained with 2% (w/v) uranylacetate in water and Reynold's lead citrate, and viewed in a Tecnai G2 Spirit BioTWIN transmission electron microscope (FEI) equipped with a Gatan Orius 200 BC CCD camera.

### 4.8 Internalisation of transferrin

HeLa cells and BSC1 cells were serum starved for 2 h prior to ligand uptake, incubated with 10 μg/mL transferrin‐Alexa546 (Life Technologies) for 5 min, washed twice with phosphate buffered saline pH 7.4 (PBS), and acid washed briefly to strip off surface‐bound ligand (0.1 M sodium acetate pH 4.6, 150 mM NaCl, 2 mM CaCl_2_). The cells were detached with trypsin, pelleted and fixed in 4% paraformaldehyde in PBS at 4°C for 10 min, pelleted and resuspended in PBS. Internalised fluorescent transferrin was quantified on a LSRFortessa cell analyser (BD Biosciences).

### 4.9 Statistical analysis

For microscopy‐based experiments where multiple experiments were analysed the number of images for each sample were chosen to provide statistically significant data for each sample (20‐50 images). For pairwise comparisons where the samples had a normal distribution an unpaired two‐sided *t* test was used, while samples where the data was not normally distributed was analysed using the Mann‐Whitney test. All statistical analysis was done using GraphPad Prism software.

## Supporting information


**Editorial Process**

**Figure S1.** Sequence alignment showing the conservation across species of the clathrin‐binding motifs in Eps15R orthologues. Alignment of Eps15R orthologues showing conservation of various DPF motifs amongst vertebrates. The numbering of residues in the consensus corresponds to positions within the human Eps15R sequence.
**Figure S2.** Eps15R clathrin‐binding mutants are not mistargeted in cells. Confocal images of HeLa cells expressing wild type EGFP‐Eps15R (A), EGFP‐Eps15R‐F703A (B), EGFP‐Eps15R‐F722A (C), EGFP‐Eps15R‐F728A (D), and endogenous immunostain of clathrin. Scale bar, 10 μm. *E,* Bar graph showing the mean size of EGFP‐Eps15R wild type and mutant punctae shown in panels A‐D. Mean ± standard error of the mean, *n* = 15 images. Student's two‐tailed unpaired *t* test
