# Supplementary material for: Eps15R and clathrin regulate EphB2‐mediated cell repulsion
Source: Traffic. 2017 Nov 6;19(1):44–57. doi: 10.1111/tra.12531 (PMC5836524; doi:10.1111/tra.12531)
Supplement: Supplementary file 2 — Figure S1. Sequence alignment showing the conservation across species of the clathrin‐binding motifs in Eps15R orthologues. Alignment of Eps15R orthologues showing conservation of various DPF motifs amongst vertebrates. The numbering of residues in the consensus corresponds to positions within the human Eps15R sequence. Figure S2. Eps15R clathrin‐binding mutants are not mistargeted in cells. Confocal images of HeLa cells expressing wild type EGFP‐Eps15R (A), EGFP‐Eps15R‐F703A (B), EGFP‐Eps15R‐F722A (C), EGFP‐Eps15R‐F728A (D), and endogenous immunostain of clathrin. Scale bar, 10 μm. E, Bar graph showing the mean size of EGFP‐Eps15R wild type and mutant punctae shown in panels A‐D. Mean ± standard error of the mean, n = 15 images. Student's two‐tailed unpaired t test [file TRA-19-44-s002.docx]

**Eps15R and clathrin regulate EphB2-mediated cell repulsion**

Emma Evergren^1, 2*^, Neville Cobbe^2^, Harvey T. McMahon^1^

^1^Medical Research Council Laboratory of Molecular Biology, Francis Crick Avenue, Cambridge CB2 0QH, UK

^2^Centre for Cancer Research and Cell Biology, Queen's University Belfast, Belfast, UK

*Corresponding author: [e.evergren@qub.ac.uk](mailto:e.evergren@qub.ac.uk)

Supplemental Materials


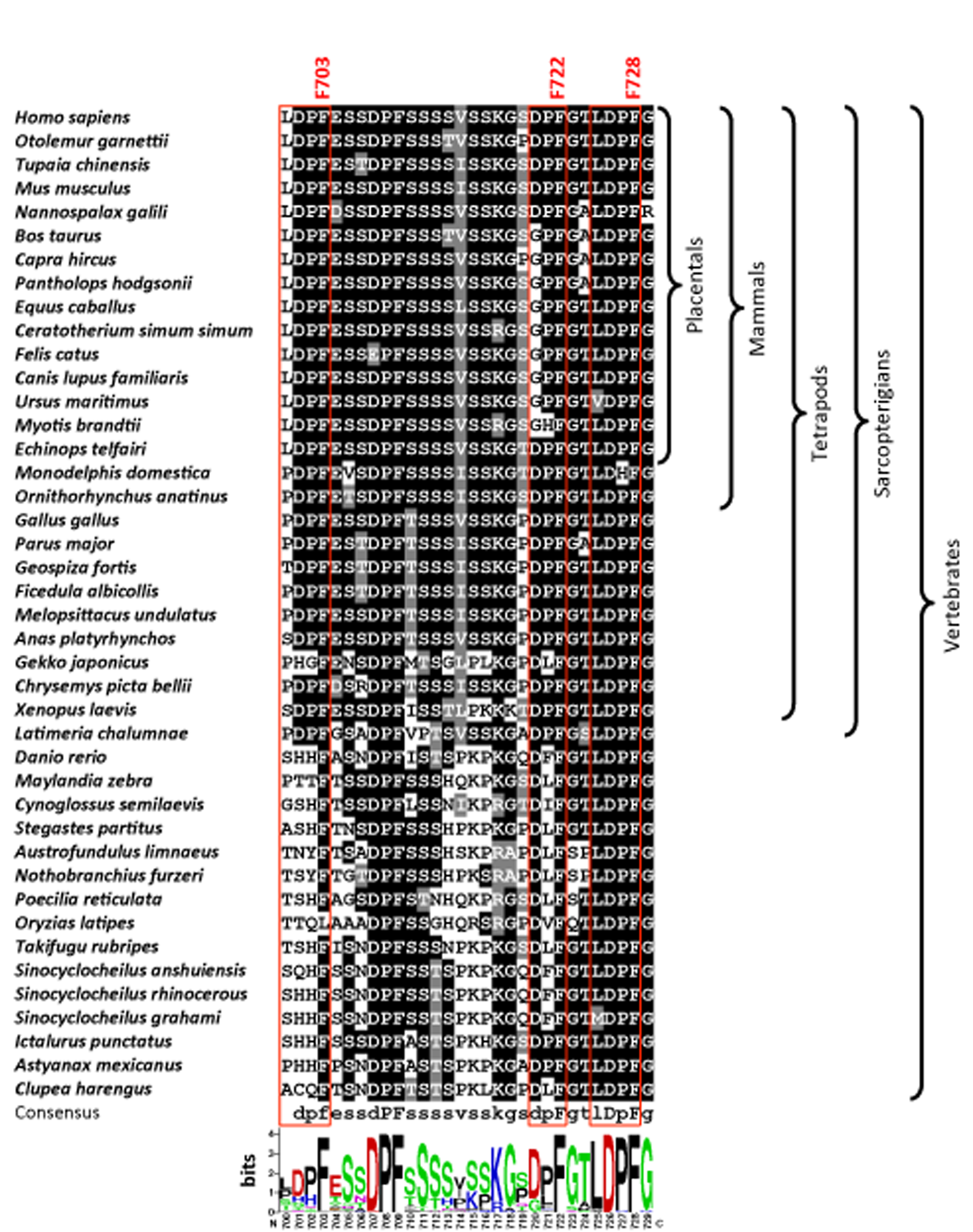


**Supplementary Figure 1. Sequence alignment showing the conservation across species of the clathrin-binding motifs in Eps15R orthologues.**

Alignment of Eps15R orthologues showing conservation of various DPF motifs amongst vertebrates.  The numbering of residues in the consensus corresponds to positions within the human Eps15R sequence.


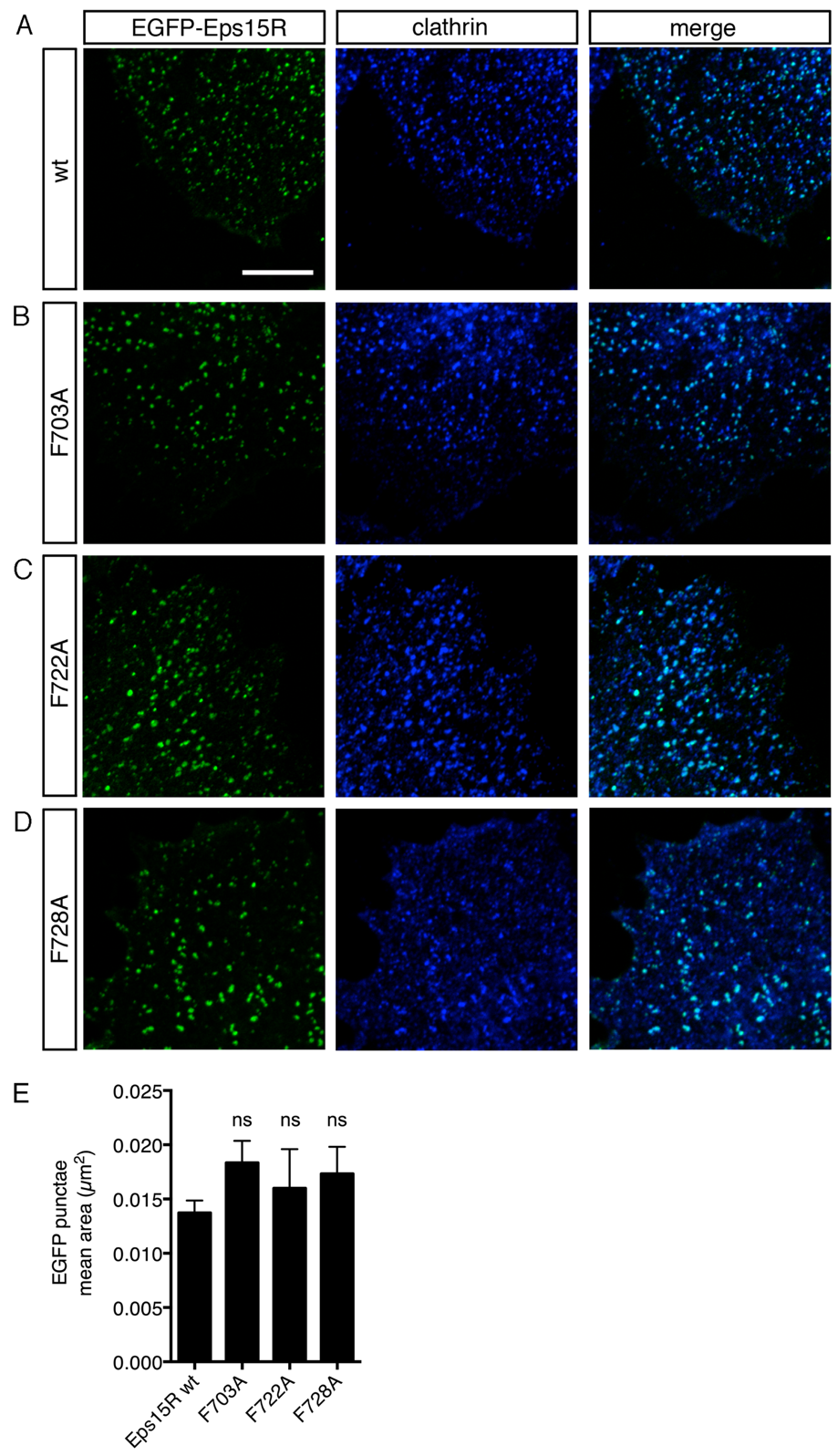


**Supplementary Figure 2. Eps15R clathrin-binding mutants are not mistargeted in cells.**

Confocal images of HeLa cells expressing wild type EGFP-Eps15R (A), EGFP-Eps15R-F703A (B), EGFP-Eps15R-F722A (C), EGFP-Eps15R-F728A (D), and endogenous immunostain of clathrin. Scale bar, 10 µm. *E,* Bar graph showing the mean size of EGFP-Eps15R wild type and mutant punctae shown in panels A-D. Mean ± standard error of the mean, n = 15 images. Student’s two-tailed unpaired t-test.
