# Supplementary material for: Eps15R and clathrin regulate EphB2‐mediated cell repulsion
Source: Traffic. 2017 Nov 6;19(1):44–57. doi: 10.1111/tra.12531 (PMC5836524; doi:10.1111/tra.12531)
Supplement: Supplementary file 1 — Editorial Process [file TRA-19-44-s001.pdf]

Title: **Eps15R and clathrin regulate EphB2-mediated cell repulsion**

Authors: Emma Evergren[1, 2*], Neville Cobbe[2], Harvey T. McMahon

Article Type: Original Research

Monitoring Editor: Alexander Sorkin
Date Submitted: 21 April 2017
Date for Decision 1: 1 May 2017
Date Resubmitted: 24 August 2017
Date for Decision 2: 20 September 2017
Accepted: 26 September 2017

Decision and Reviews

Dear Dr. Evergren

Thank you for submitting your manuscript "Eps15R and clathrin regulate EphB2-mediated cell repulsion" for consideration for publication in Traffic. I asked two colleagues who are experts in the field to review the paper and their verbatim comments are appended below. The referees share the view that the work presented in this paper will be of interest to the readers of Traffic, but that additional experiments are necessary to more fully support your conclusions. In particular, it is important to address the issue of the redundancy of Eps15R and Eps15. Also, clear presentation and careful quantification of the results of imaging, western blotting and repulsion assays would strengthen the manuscript. In addition, referee 1 notes that supplemental figures 1 and 3 may not be necessary.

Although I cannot accept your manuscript for publication at this point, I believe that you will be able to address the referees concerns and I look forward to receiving your revised manuscript. To expedite handling when you resubmit please be sure to include a response outlining how you have addressed each of the referees' concerns.

Sincerely,

Alexander Sorkin, Ph.D.
Associate Editor

Referee's Comments to the Authors

Referee: 1

Comments to the Author
Authors aim to investigate the role of clathrin-mediated endocytosis in EphB2-mediated cell repulsion. This is an area of research that is certainly understudied. Authors also present data regarding the role of Eps15R in clathrin-dependent endocytosis and identify a clathrin-binding motif in Eps15R.
The narrative as presented is confusing. It starts with EphB2, presents a lot of data about Eps15R then goes back to EphB2. In my opinion, it would make more sense to start with the assessment of the role of Eps15R in clathrin-dependent endocytosis, warranted based on the presence of some controversy regarding this role, then would show data related to EphB2, as a proof of concept. This would make more sense because Eps15R rather than EphB2 seems to be the central point of the work (unlike what the title indicates).

The role of Eps15R in clathrin-dependent endocytosis of EphB2-EphrinB1 complexes relies too heavily on the cell mixing assay. In view of the large variability of this assay (fig 1 and 7) A more direct analysis (e.g. immunofluorescence) is needed to provide a more mature assessment.

In more details, here are my suggestions to make the manuscript more suitable for publication:
1) Fig 1A,B: the method of calculation of the % of EGFP+cells in clusters should be better described, in particular how are "clusters" defined. As a suggestion, a more quantitative method could be the inclusion of a nuclear staining (e.g. DAPI on fixed cells or Hoechst 33342 dye for viable cells to minimize loss of cells and disaggregation of clusters due to fixation and washes) to obtain a more accurate number of cells per cluster. This could bypass the need for the use of the ImageJ macro which, from the comparison

between fig1B and fig1C, appears to reflect less the extent of differences observed in fig1A than the cluster measurement method. The large SDs in fig 7 also question the quantitative nature of the method. In summary, the adaptation of clonalTool to the study of cell-cell repulsion, although very interesting, might need some optimization.

2) Fig 1: The legend mentions that "A Coomassie stained SDS -PAGE gels shows the equal loading of the EH domains". Is this "data not shown" or is the panel missing? Myc-Eps15R is also precipitated in absence of Flag-EphB2 transfection (first left well in the blot). Any explanation for this? The quality of the blot precludes from making a quantitative conclusion such as that "The interaction was more prominent upon stimulation of the EphB2-expressing cells with pre-clustered extracellular domain of ephrinB1". Please provide a better blot. Furthermore, stimulation can only be shown if EphB2 phosphorylation or activation of a downstream signaling is assessed.

3) The rationale behind Supplementary Figure 1 and the need to generate a phylogenetic tree showing the evolutionary divergence of Eps15R, Eps15 and intersectin, is not clear in the context of this work.

4) Fig 4E: Could the DPF sequence around F709 and which is also conserved be involved?.

5) Fig 4G: the pull-down assay lacks a full length wild type clathrin control for maximum interaction. The related comment in the text reads: "Only the terminal domain of clathrin bound Eps15R efficiently (Fig. 4G).", and should specify "N-terminal".

6) Fig 5 and 6: There seems to be a difference in the size of the punctate signals in single mutants (Fig 5 panels A/B versus C/D) that is more pronounced than between wt and F703A/F722A/F728A triple mutant (Fig 6). Is there any explanation? Was any quantitative analysis performed on single mutants? It would make more sense if single and triple mutants were combined in the same figure. Also, please perform statistical analysis for Fig6 B and D.

7) Fig 7: Control immunoblots showing the efficiency of shRNA knock down and rescue, would improve the results.

Referee: 2

Comments to the Author
The interaction between EphB receptors and the ephrinB ligand in neighboring cells leads to the internalization of EphB from the plasma membrane, a process termed trans-endocytosis and is required for cell repulsion and migration. In this manuscript, the authors use a colorectal cancer cell line to visualize and assess repulsion, and find evidence that it relies upon clathrin-dependent endocytosis of EphB2. Visualization was done using an EphrinB1-RFP/EphB2-GFP co-culture system with an automated program to calculate the degree of clustering as a measure of repulsion, and using a mutant dynamin and C-terminal AP180 fragment to inhibit internalisation. The authors then found that NPF motifs of the endocytic adaptor protein numb bound selectively to the second of 3 EH domains in both Eps15 and Eps15R (but not of intersectins 1 and 2), and that a protein complex comprised of EphB2-numb and Eps15R serves as an adaptor complex in clathrin-mediated internalization of EphB2. The authors then tested the kinetics of clathrin-coated pit formation in the absence of Eps15R, and found that the rate of CCP formation is decreased. Through a series of GST-binding assays, they found that clathrin heavy chain binds directly to Eps15R via a DPFxxLDPF motif. However, perhaps somewhat surprisingly, mutating each of the key phenylalanines in the Eps15R motif that binds to clathrin had no effect on the recruitment/localization of clathrin to punctae at the plasma membrane. These findings led the authors to a series of experiments promoting the notion that Eps15R regulates the interaction between clathrin and AP-2, suggesting overall that Eps15R does not regulate clathrin recruitment, but does control CCP formation. Finally, the authors knocked down Eps15R and assayed repulsion, finding smaller EphB2 clusters, indicative of decreased repulsion. This could be rescued with wtEps15R, but not with the clathrin-binding Eps15R mutant, providing evidence for the Eps15R-clathrin interaction as being important for trans-endocytosis.

This is an interesting manuscript that addresses the function of Eps15R and clathrin in trans-endocytosis and cell-cell repulsion. Overall, the experimental data is generally convincing and controlled, and the conclusions are sound. The primary concern is that the manuscript ignores the potential interplay between the two related proteins, Eps15 and Eps15R, exclusively focusing on the latter. This not only narrows the focus of the manuscript, but may lead to some confusion, as in many cases these proteins may be functioning together or compensating for each other. Addressing both proteins would make the manuscript considerably sounder, more interesting, and useful to the reading audience.

Specific Concerns:
1.) It is unclear why the authors chose to exclusively pursue the involvement of Eps15R as a part of the adaptor complex involved in EphB2 internalization. Indeed, they showed that in vitro, both Eps15 and Eps15R interact equally well with numb, so the rationale for ignoring Eps15 remains unclear. Do these two proteins play similar/compensatory roles in EphB2 endocytosis? Or do they compete for numb and antagonize each others' functions? The fact that Eps15R has been studied less and the two proteins are evolutionarily divergent is not a strong rationale for focusing exclusively on Eps15R.

2.) A related concern lies in the finding that Eps15R knock-down apparently leads to a slowing down of clathrin coated pit formation, as measured by the lifetime of GFP-tagged AP-2sigma2 containing punctae. How does Eps15 compensate for this knock-down? Is it possible that due to overlapping functions, that in cells expressing higher Eps15 levels the Eps15R knock-down has a more limited effect? What happens of both proteins are knocked down simultaneously?

3.) The individual alanine mutants for Eps15R 703, 722 and 728 do not reduce affinity for clathrin sufficiently to affect the latter's recruitment to the PM; why have the authors not tried a triple mutant with 703/722/728?

4.) Are the DPF motifs in Eps15 homologous to those in Eps15R, and do they also bind directly to clathrin? Would KD of both proteins prevent recruitment of clathrin to the PM?

5.) Some of the representative images provided for the repulsion assays are not especially compelling. While it is recognized that the authors quantified multiple images at many regions, it might be helpful to provide more clear cut examples of repulsion along with the data for those individual micrographs, so that readers can appreciate the differences and the potency of the assay.

Author Rebuttal

Dear Editor,
We would like to thank you and the peer reviewers for the positive and constructive feedback on our manuscript. Your helpful summary points have been addressed in full as have all of the points raised by the two peer reviewers. In overview we have addressed the issue of redundancy of Eps15R and Eps15 by confirming that knockdown of Eps15R does not alter Eps15 expression and yet has significant impacts on clathin-coated pit lifetime – a phenotoypic effect that is not achieved by Eps15 knockdown (Figure 3A-C). Biochemically Eps15R interacts with clathrin through direct binding to short amino acid motifs, which are not present in Eps15 (Figure 4F). Furthermore the phenotypic effect of Eps15R knockdown can be rescued by Eps15R overexpression but not by Eps15 (Figure 6B) as described in the original submission and now further confirmed through additional experiments and quantitation. The figures and the quantification of data have been enhanced with more experimental replicates and restructuring of data presentation. This restructuring has included, as requested, the removal of supplemental figures 1 and 3. This restructuring has also led to a reduction in the number of main figures by switching figure 5 in the primary submission to the supplementary information. Figure 5 represented imaging on single mutants within the clathrin-binding region. Prompted by the reviewers further quantitative analysis proved that these single mutants were not significant in cells unlike the triple mutant. This has significantly improved the manuscript. We enclose a revised main text file with text changes highlighted in yellow. A detailed response to the reviewers comments can be found in the cover letter, which includes embedded images.

Yours sincerely,
Emma Evergren

Decision and Reviews

Dear Dr. Evergren,

Thank you for submitting your revised manuscript "Eps15R and clathrin regulate EphB2-mediated cell repulsion" to Traffic. I asked the referees to read the revised paper and their verbatim comments are appended below. I agree with the referees that you have addressed the concerns raised previously. Referee 1 notes that the paper needs careful editing before the paper can be accepted. Please be sure to check the entirety of the text, not just the highlighted concerns. With these changes, I would be pleased to accept this paper for publication in Traffic.

Sincerely,

Alexander Sorkin, Ph.D.
Associate Editor

Referee's Comments to the Authors

Referee: 2

Comments to the Author
The authors have satisfactorily addressed my concerns from the previous submission.

Referee: 1

Comments to the Author
This version of the manuscript is neatly improved. Thanks to the authors for the significant effort made particularly with regard to additional controls, quantification of the data and re-organization.
There are just few minor errors. For instance there is redundancy in the Synopsis: "EphB receptors and their ligands are important during formation of tissue boundary formation during development"
Few mistakes, for example on page 5: "To investigate weather the Eps15R"(Whether)
Please double-check uniformity of referencing style. Page 7: " (Scheele JBC 2003)."
